# Transgenic up-regulation of alpha-CaMKII in forebrain leads to increased anxiety-like behaviors and aggression

**DOI:** 10.1186/1756-6606-2-6

**Published:** 2009-03-04

**Authors:** Shunsuke Hasegawa, Takahiro Furuichi, Taro Yoshida, Kengo Endoh, Kenichi Kato, Megumi Sado, Ryouta Maeda, Aya Kitamoto, Takahisa Miyao, Ryosuke Suzuki, Seiichi Homma, Shoichi Masushige, Yasushi Kajii, Satoshi Kida

**Affiliations:** 1 Department of Bioscience, Faculty of Applied Bioscience, Tokyo University of Agriculture, Tokyo 156-8502, Japan; 2 Pharmacology Laboratory Mitsubishi Tanabe Pharma Corporation, Yokohama 227-0033, Japan; 3 Core Research for Evolutional Science and Technology, Japan Science and Technology Agency, Saitama 332-0012, Japan

## Abstract

**Background:**

Previous studies have demonstrated essential roles for alpha-calcium/calmodulin-dependent protein kinase II (alpha-CaMKII) in learning, memory and long-term potentiation (LTP). However, previous studies have also shown that alpha-CaMKII (+/-) heterozygous knockout mice display a dramatic decrease in anxiety-like and fearful behaviors, and an increase in defensive aggression. These findings indicated that alpha-CaMKII is important not only for learning and memory but also for emotional behaviors. In this study, to understand the roles of alpha-CaMKII in emotional behavior, we generated transgenic mice overexpressing alpha-CaMKII in the forebrain and analyzed their behavioral phenotypes.

**Results:**

We generated transgenic mice overexpressing alpha-CaMKII in the forebrain under the control of the alpha-CaMKII promoter. In contrast to alpha-CaMKII (+/-) heterozygous knockout mice, alpha-CaMKII overexpressing mice display an increase in anxiety-like behaviors in open field, elevated zero maze, light-dark transition and social interaction tests, and a decrease in locomotor activity in their home cages and novel environments; these phenotypes were the opposite to those observed in alpha-CaMKII (+/-) heterozygous knockout mice. In addition, similarly with alpha-CaMKII (+/-) heterozygous knockout mice, alpha-CaMKII overexpressing mice display an increase in aggression. However, in contrast to the increase in defensive aggression observed in alpha-CaMKII (+/-) heterozygous knockout mice, alpha-CaMKII overexpressing mice display an increase in offensive aggression.

**Conclusion:**

Up-regulation of alpha-CaMKII expression in the forebrain leads to an increase in anxiety-like behaviors and offensive aggression. From the comparisons with previous findings, we suggest that the expression levels of alpha-CaMKII are associated with the state of emotion; the expression level of alpha-CaMKII positively correlates with the anxiety state and strongly affects aggressive behavior.

## Background

Alpha-CaMKII is a Ser/Thr protein kinase that is abundantly expressed in the forebrain [1-5]. In response to an increase in the intracellular concentration of Ca^2+^, alpha-CaMKII is activated through the binding of Ca^2+^/calmodulin (CaM), and then phosphorylates target proteins to activate or inactivate these proteins [1-5]. Furthermore, the prolonged activation of alpha-CaMKII by Ca^2+^/CaM results in the intramolecular autophosphorylation of Thr residues such as T286, T305, and T306 [1-5]. Autophosphorylation of T286 leads to a decrease in the dissociation of bound Ca^2+^/CaM and continuous partial activation even after the dissociation of Ca^2+^/CaM, thereby prolonging its activation. Thus, a transient increase in intracellular Ca^2+ ^can result in prolonged alpha-CaMKII activation until T286 is dephosphorylated by protein phosphatases [1-5].

Genetic studies have examined the effects of the loss or gain of function of alpha-CaMKII [2-15]. Genetic deletion of alpha-CaMKII impaired hippocampus-dependent spatial learning in homozygous knockout mice [8]. Furthermore, mice with a Thr286 to Ala (T286A) point mutation leading to a lack of the autonomous activity of this kinase have also shown severe impairments of spatial learning [9]. These findings indicate that alpha-CaMKII is an essential molecule involved in learning and memory. However, several studies have shown that overexpression of alpha-CaMKII or an increase in alpha-CaMKII activity does not simply lead to an enhanced ability in learning/memory [11-15]. Transgenic mice overexpressing wild-type or a constitutively active mutant (T286D) of alpha-CaMKII in the forebrain showed deficits in learning/memory [11-15].

Interestingly, a previous study also showed that mutant mice with a deletion of the alpha-CaMKII gene display abnormal emotional behavior [16]. Both alpha-CaMKII (-/-) homozygous and (+/-) heterozygous knockout mice exhibited attenuated fear behavior. Furthermore, heterozygous knockout mice showed increased defensive aggression but normal offensive aggression [16], which is consistent with a reduction in fear behavior. A recent study extended these observations; heterozygous knockout mice displayed a dramatic decrease in anxiety-like behaviors and symptoms of psychiatric disorders such as bipolar disorders and schizophrenia [17]. These findings indicate that alpha-CaMKII plays critical roles in not only learning and memory, but also in emotional behaviors including anxiety, the fear response and aggression [16,17].

In this study, to understand the role of alpha-CaMKII in emotional behavior, we generated transgenic mice expressing wild-type alpha-CaMKII in the forebrain under the control of the alpha-CaMKII promoter. These transgenic mice displayed an increase in anxiety-like behavior and a decrease in locomotor activity, which are the opposite phenotypes to those observed in alpha-CaMKII (+/-) heterozygous knockout mice [16,17]. Interestingly, in contrast to the observations in alpha-CaMKII (+/-) heterozygous knockout mice, alpha-CaMKII overexpressing mice exhibited increased offensive but normal defensive aggression. Taken together with previous observations, our findings suggest that the expression level of alpha-CaMKII plays important roles in emotional behavior, and especially, that the expression level of alpha-CaMKII positively correlates with anxiety-like behaviors.

## Results

### Generation of alpha-CaMKII overexpressing mice

**Figure 1:**
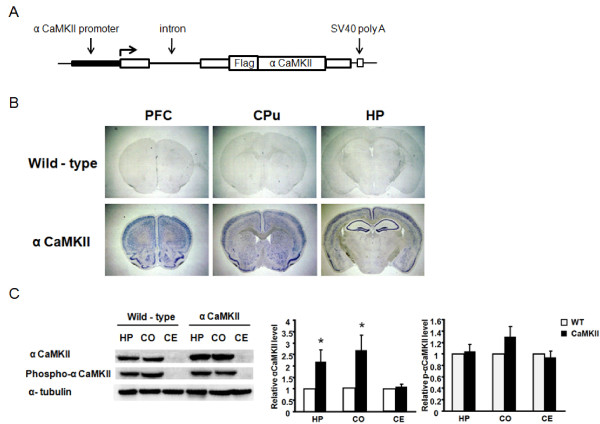
**Generation and expression analyses of alpha-CaMKII overexpressing mice**. (A) Schematic representation of the alpha-CaMKII transgenic construct. (B) *In situ *hybridization analyses of transgene mRNA expression in the alpha-CaMKII overexpressing and WT mice. (C) Western blot analyses of the levels of alpha-CaMKII and phosphorylated alpha-CaMKII at T286 in the hippocampus, cortex, and cerebellum of alpha-CaMKII overexpressing and WT mice. Levels of alpha-CaMKII and phosphorylated alpha-CaMKII were normalized according to the levels of alpha-tubulin (hippocampus, WT, n = 7; alpha-CaMKII, n = 7; cortex, WT, n = 7; alpha-CaMKII, n = 7; cerebellum, WT, n = 5; alpha-CaMKII, n = 5). The asterisk indicates statistical significance at P < 0.05.

To investigate the role of alpha-CaMKII in emotional behavior, we generated transgenic mice overexpressing wild-type alpha-CaMKII in the forebrain using the alpha-CaMKII promoter [12-14,18,19]. We first constructed a transgene that contained the alpha-CaMKII promoter, a hybrid intron in the 5'-untranslated leader sequence, the coding region of alpha-CaMKII fused with the FLAG-tag sequence at the N-terminus, and a polyadenylation signal (Figure 1A). The alpha-CaMKII promoter used in this study has been known to exhibit strong activity in regions of the forebrain including the hippocampus, cortex and striatum [12-14,18,19].

We next performed expression analyses in the transgenic alpha-CaMKII mice. *In situ *hybridization analyses revealed high levels of expression of transgene mRNA in regions of the forebrain including the CA1, CA3, and dentate gyrus areas of the hippocampus, basolateral and lateral areas of amygdala and cortical and prefrontal regions (Figure 1B). To compare the expression level of alpha-CaMKII between transgenic and wild-type (WT) littermates, we analyzed the levels of alpha-CaMKII in the forebrain using Western blotting. Quantitative immunoblotting with antibodies recognizing alpha-CaMKII revealed 2–2.5 fold higher expression of alpha-CaMKII in the hippocampus and cortex of transgenic mice compared with WT littermates (HP, F_1,12 _= 5.513, P = 0.0368; CO, F_1,12 _= 6.382, P = 0.0266; Figure 1C). Interestingly, no difference was observed in the level of the phosphorylated form of alpha-CaMKII at T286 between the alpha-CaMKII overexpressing mice and WT littermates (Figure 1C). This is a similar observation with previous findings in which alpha-CaMKII (+/-) heterozygous knockout mice display comparable levels of phosphorylated alpha-CaMKII at T286 with WT littermates [17]. Some compensatory mechanisms might exist by which the activity of alpha-CaMKII is kept at the constant basal level.

Previous study has shown that alpha-CaMKII (+/-) heterozygous knockout mice displayed abnormalities of gene expression profiles in hippocampus [17]. To examine effects of overexpression of alpha-CaMKII at the molecular level, we analyzed hippocampal expression levels of five genes that decreased in hippocampus of alpha-CaMKII (+/-) heterozygous knockout mice (Additional file 1, Figure S1). Analyses using quantitative PCR showed that alpha-CaMKII overexpressing mice exhibited a significant decrease in nephronectin (Npnt) mRNA level (F_1,8 _= 6.409, P = 0.0352; Additional file 1, Figure S1A) and trends towards to a decrease in mRNA levels of tryptophan 2, 3-dioxygenase (Tdo; F_1,8 _= 3.99, P = 0.0808; Additional file 1, Figure S1B) and solute carrier family 39 (metal ion transporter) member 6 (Slc39a6; F_1,8 _= 1.902, P = 0.2052; Additional file 1, Figure S1C) in the hippocampus compared with WT mice. Thus alpha-CaMKII overexpressing mice displayed similar changes in gene expressions with alpha-CaMKII (+/-) heterozygous knockout mice although these alterations of gene expression in alpha-CaMKII overexpression mice were not dramatic compared with those in alpha-CaMKII (+/-) heterozygous knockout mice (See 17). Further studies are required to understand the relationships between changes in these gene expressions and hippocampal function in mutant mice showing down or up regulation of alpha CaMKII level in hippocampus.

### Increased anxiety-like behavior in alpha-CaMKII overexpressing mice

Previous studies have demonstrated that alpha-CaMKII (+/-) heterozygous knockout mice display a dramatic decrease in anxiety-like behaviors [16,17]. Therefore, to understand the roles of alpha-CaMKII in anxiety-like behaviors, we performed open field, elevated zero maze, light-dark transition and social interaction tests, which enabled us to estimate the anxiety state of alpha-CaMKII overexpressing mice.

**Figure 2:**
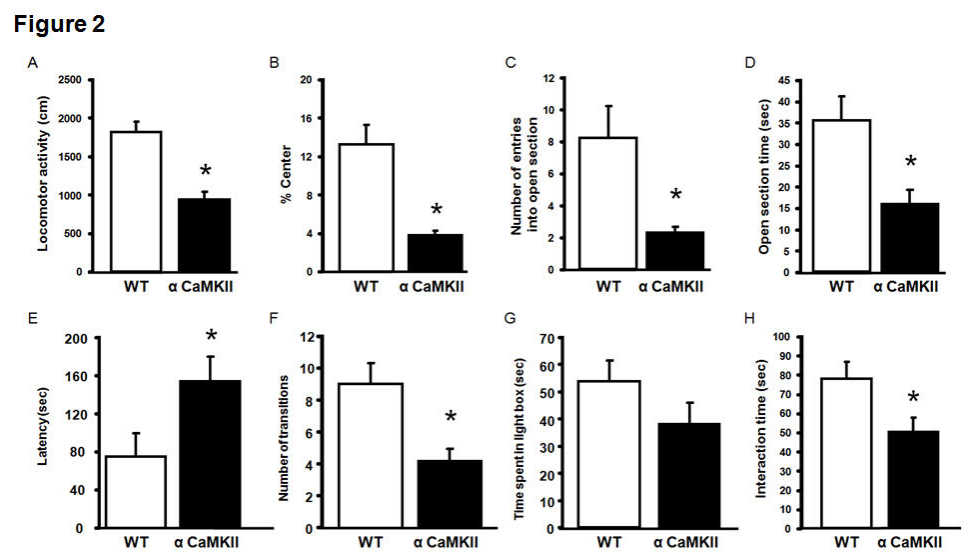
**alpha-CaMKII overexpressing mice exhibit an increase in anxiety-like behaviors**. (A, B) The total path length (A) and the percent of time spent in the center (B) the open field test, are shown for alpha-CaMKII overexpressing (n = 10) and WT mice (n = 10). (C, D) The number of entries into the open section (C) and the time spent in the open section (D) in the elevated zero maze test, are shown for alpha-CaMKII (n = 26) and WT mice (n = 13). (E-G) Latency (E), number of transitions (F) and time spent in light box (G) in light/dark transition test, are shown for alpha-CaMKII (n = 13) and WT mice (n = 13). (H) Social interaction time in the social interaction test are shown for alpha-CaMKII (n = 13) and WT mice (n = 12). The asterisk indicates statistical significance at P < 0.05.

We first performed the open field test to measure anxiety-like behavior and locomotor activity. Since mice fear novel and open spaces, and generally avoid the center of open fields, this test is used to measure anxiety. alpha-CaMKII overexpressing mice exhibited a significantly reduced total path in the open field compared to WT littermates (F_1,26 _= 26.79, P < 0.0001; Figure 2A). More importantly, these mutant mice exhibited a significant decrease in the percentage of paths in the center of the field (F_1,26 _= 24.206, P < 0.0001; Figure 2B). These results suggest that alpha-CaMKII overexpressing mice display a decrease in locomotor activity in a novel environment and an increase in anxiety-like behavior.

We next performed the elevated zero maze test. In this task, since mice generally avoid high open spaces, the duration of time that they spend in the open spaces and the number of times they enter such spaces are thought to reflect their state of anxiety. Similarly with the results in the open field test, alpha-CaMKII overexpressing mice exhibited significantly decreased number of entries into the open section (F_1,37 _= 15.754, P = 0.0003; Figure 2C) and time spent in the open section (F_1,37 _= 9.190, P = 0.0044; Figure 2D) compared with WT mice. These results are consistent with the previous observations in the open field test (Figure 2A, Figure 2B) and suggest that alpha-CaMKII overexpressing mice exhibited a significant increase in anxiety-like behavior. We next performed the light-dark transition test. Since mice generally avoid light spaces, the latency until the mice enter the light box from the dark box, the duration that they spend in the light box and the number of transitions they make from the dark box to the light box are thought to reflect their state of anxiety. alpha-CaMKII overexpressing mice exhibited a significant increase in the latency until they entered the light box (F_1,24 _= 4.941, P = 0.0359; Figure 2E) and a significant decrease in the number of transitions they made (F_1,24 _= 9.599, P = 0.049; Figure 2F) compared with WT mice. In addition, we also observed a trend towards to a decrease in the time that alpha-CaMKII overexpressing mice spent in the light box (F_1,24 _= 1.981, P = 0.1721; Figure 2G). Thus, these changes suggest that the up-regulation of alpha-CaMKII expression leads to an increase in anxiety.

An increase in the state of anxiety is thought to lead to a decrease of social behavior such as social investigation [20]. Therefore, we finally measured the social interactions of male alpha-CaMKII overexpressing mice with juvenile male mice. These transgenic mice displayed a significantly shorter social interaction time with juvenile mice compared with WT mice during a 3 min social interaction test (F_1,23 _= 6.379, P = 0.0189; Figure 2H). This result indicates that alpha-CaMKII overexpressing mice display a decrease in social behavior. This decrease in social behavior might reflect the increase in anxiety-related behavior.

Collectively, all of these observations suggest that up-regulation of alpha-CaMKII expression in the forebrain leads to an increase in anxiety. Interestingly, these phenotypes of anxiety-like behaviors observed in alpha-CaMKII overexpressing mice are the opposite to those observed in alpha-CaMKII (+/-) heterozygous knockout mice that display a dramatic decrease in anxiety-like behaviors [16,17]. Therefore, these comparisons between alpha-CaMKII overexpressing and alpha-CaMKII (+/-) heterozygous knockout mice suggest that the expression level of alpha-CaMKII positively correlates with the anxiety state; more alpha-CaMKII expression leads to an increase in anxiety.

### Decreased locomotor activity of alpha-CaMKII overexpressing mice in their home cages

**Figure 3:**
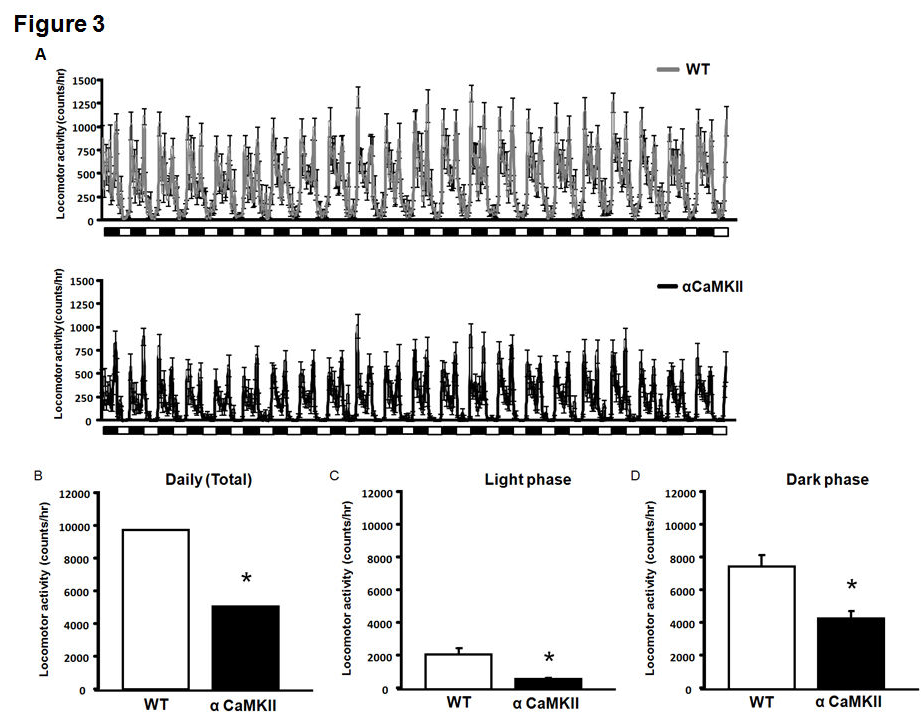
**alpha-CaMKII overexpressing mice exhibit a decrease in locomotor activity in their home cages**. (A-B) Changes in locomotor activity of WT (A) and alpha-CaMKII overexpressing (B) in their home cages. (C-E) Locomotor activity during a day (C) and light (D) and dark (E) phases are shown for alpha-CaMKII overexpressing (n = 11) and WT mice (n = 13). The asterisk indicates statistical significance at P < 0.05.

A recent study has shown that alpha-CaMKII (+/-) heterozygous knockout mice display increased locomotor activity and periodic mood-change-like behavior in their home cages [17]. Our results indicate that alpha-CaMKII overexpressing mice display a decrease in locomotor activity in a novel environment (Figure 2A). Therefore, to examine whether this decrease in locomotor activity observed in the alpha-CaMKII overexpressing mice is specific for novel environments, we measured the locomotor activity of alpha-CaMKII overexpressing mice in their home cages. In contrast to the results observed for alpha-CaMKII (+/-) heterozygous knockout mice, alpha-CaMKII overexpressing mice exhibited a significant decrease in locomotor activity in their home cages during the day and in both the light and dark phases (day, F_1,22 _= 17.419, P = 0.0004; light phase, F_1,22 _= 15.085 P = 0.0008; dark phase, F_1,22 _= 14.071, P = 0.0011; Figure 3). These results suggest that the decrease in locomotor activity observed in alpha-CaMKII overexpressing mice in the open field test is not due to the response to the novel environment. These contrasting observations between alpha-CaMKII (+/-) heterozygous knockout and overexpressing mice suggest that the expression level of alpha-CaMKII negatively correlates with locomotor activity.

### Motor coordination of alpha-CaMKII overexpressing mice

**Figure 4:**
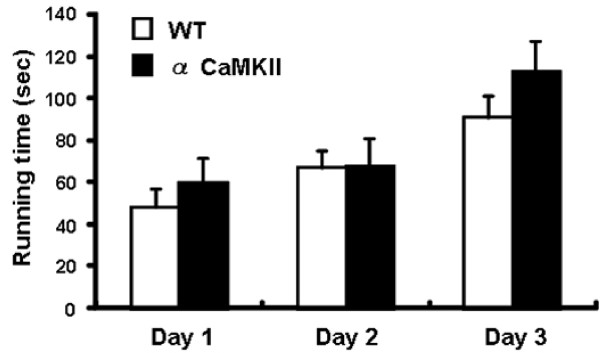
**alpha-CaMKII overexpressing mice exhibit normal motor performance in the rotarod test**. Running time is shown for alpha-CaMKII overexpressing (n = 13) and WT mice (n = 16).

It is possible that the abnormal emotional behaviors, especially the decrease in locomotor activity in a novel environment and their home cages observed in alpha-CaMKII overexpressing mice is due to impaired motor performance. To examine this possibility, we subjected mice to the accelerating rotarod test. Our results showed that alpha-CaMKII overexpressing and WT mice displayed a comparable degree of motor performance (day 1, F_1,27 _= 0.719, P = 0.4041; day 2, F_1,27 _= 0.006 P = 0.9369; day 3, F_1,27 _= 1.45, P = 0.239; Figure 4), suggesting that up-regulation of alpha-CaMKII expression in the forebrain did not affect motor coordination. Thus, the decrease in locomotor activity observed in alpha-CaMKII overexpressing mice was not likely to have been due to decreased motor performance.

### Increased offensive aggression in alpha-CaMKII overexpressing mice

**Figure 5:**
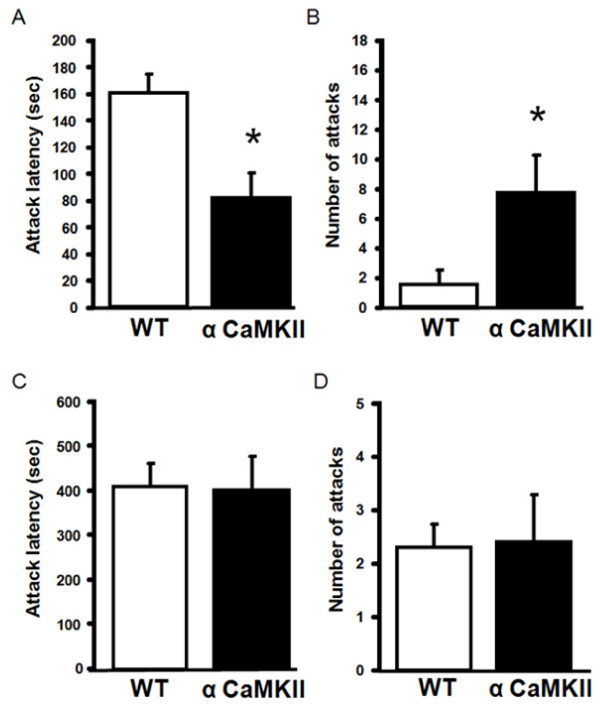
**alpha-CaMKII overexpressing mice display an increase in offensive aggression in the resident-intruder test**. (A, B) offensive aggression. (C, D) defensive aggression. The latency to first attack bite (attack latency; A, C) and the number of attacks (B, D) are shown for alpha-CaMKII overexpressing (n = 10) and WT mice (n = 10). The asterisk indicates statistical significance at P < 0.05.

Previous studies have demonstrated that alpha-CaMKII (+/-) heterozygous knockout mice display increased defensive aggression but normal offensive aggression [16]. Interestingly, alpha-CaMKII overexpressing mice also showed increased aggression towards their cage mates. Therefore, to further understand the role of alpha-CaMKII in aggressive behavior, we performed the resident-intruder test. To assess offensive aggression, resident mice were individually housed for 4 weeks prior to the introduction of a WT male intruder mouse that had previously been housed in a group of five animals. alpha-CaMKII overexpressing resident mice showed significantly higher offensive aggression, as assessed by their attack latency (F_1,24 _= 9.939, P = 0.0043; Figure 5A) and number of attacks (F _1,24 _= 4.463, P = 0.0452; Figure 5B), compared with WT resident mice. To assess defensive aggression, mice that had previously played the role of a resident animal became the intruder; a different group of WT mice that had been individually housed for 4 weeks, were used as the residents. alpha-CaMKII overexpressing intruder mice showed a comparable attack latency (F _1,18 _= 0.01, P = 0.92; Figure 5C) and number of attacks (F _1,18 _= 0.01, P = 0.9208; Figure 5D) with WT intruder mice, indicating that alpha-CaMKII overexpressing mice display normal defensive aggression. These results indicate that in contrast to alpha-CaMKII (+/-) heterozygous knockout mice, alpha-CaMKII overexpressing mice display increased offensive aggression but normal defensive aggression.

## Discussion

**Figure 6:**
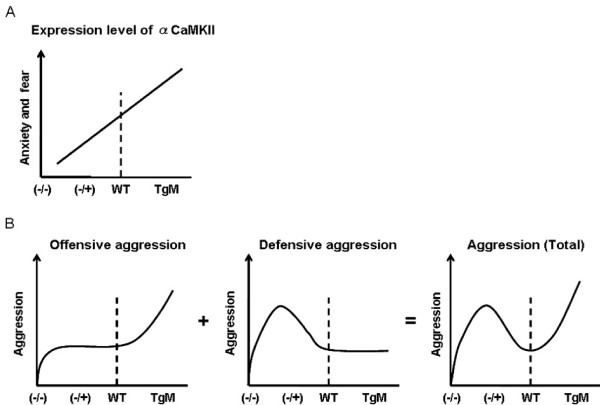
**The relationship between the expression level of alpha-CaMKII in the forebrain and anxiety, fear and aggression**. (A) Expression level of alpha-CaMKII positively correlates with anxiety-like and fearful behaviors. (B) alpha-CaMKII (+/-) heterozygous knockout and alpha-CaMKII overexpressing mice display an increase in defensive and offensive aggression, respectively.

Alpha-CaMKII is believed to play essential roles in synaptic plasticity and learning/memory [1-5]. However, previous studies have demonstrated that alpha-CaMKII (+/-) heterozygous knockout mice display a dramatic decrease in fear behavior and anxiety-like behavior, and increased defensive aggression but normal offensive aggression [16,17]. These findings indicate that alpha-CaMKII regulates multiple brain functions including not only learning/memory but also emotional behaviors. In this study, to further understand the roles of alpha-CaMKII in emotional behavior, we generated transgenic mice overexpressing alpha-CaMKII in the forebrain and analyzed the behavioral phenotypes of these transgenic mice. In contrast to the behavioral phenotypes observed in alpha-CaMKII (+/-) heterozygous knockout mice, alpha-CaMKII overexpressing mice exhibited a significant increase in anxiety-like behaviors in the open field, elevated zero maze and light-dark transition tests, suggesting that up-regulation of alpha-CaMKII expression leads to an increase in anxiety. Furthermore, these overexpressing mice also display increased offensive aggression but normal defensive aggression. Therefore, taken together with previous observations [16,17], our findings indicate that the expression level of alpha-CaMKII has a strong impact on emotional behavior, especially anxiety-like behavior and aggression (Figure 6).

alpha-CaMKII is believed to be an essential molecule for learning/memory [1-5]. Indeed, alpha-CaMKII (-/-) homozygous knockout mice showed deficits in learning [8]. Furthermore, mutant mice lacking the autonomous activity of alpha-CaMKII (T286A mutant mice) also exhibited impaired learning [9]. However, up-regulation of alpha-CaMKII expression or activity does not simply result in the expected gain of function-effects such as the enhancement of leaning; transgenic mice overexpressing a constitutively active mutation of alpha-CaMKII (T286D) or wild-type alpha-CaMKII in the forebrain exhibited impaired learning and memory formation [11-15]. Consistent with previous studies [14], we also observed that the alpha-CaMKII overexpressing mice analyzed in this study displayed deficits in memory formation (data not shown). These observations are in contrast to the case of CaMKIV which positively regulates memory formation; a null mutation of the CaMKIV gene impairs memory consolidation [21-23], while forebrain-specific overexpression of this kinase enhances memory consolidation [19,24]. These previous studies suggest that alpha-CaMKII levels do not simply correlate with learning/memory performance [14,15]. However, we cannot exclude other possibilities; a moderate up-regulation of alpha-CaMKII expression levels or activity is required for the enhancement of learning/memory. Additionally, transgenes used in our and other studies lack 3'-untranslated region of alpha-CaMKII targeting signals for the localization of alpha-CaMKII mRNA to the dendrites [11-15,25]. Therefore, the possibility still remains that this lack of 3'UTR of alpha-CaMKII in transgenes resulted in abnormal intracellular localization of overexpressed alpha-CaMKII in cell bodies, thereby leading to deficits in learning/memory and abnormal emotional behaviors. In contrast, alpha-CaMKII overexpressing mice and alpha-CaMKII (+/-) heterozygous knockout mice displayed opposite emotional behavioral phenotypes; alpha-CaMKII overexpressing mice displayed an increase in anxiety-like behavior and a decrease in locomotor activity, while alpha-CaMKII (+/-) heterozygous knockout mice display a decrease in anxiety-like behavior and an increase in locomotor activity [16,17]. Therefore, these comparisons strongly suggest that the expression level of alpha-CaMKII might determine the emotional state, and especially that the expression level of alpha-CaMKII positively correlates with the state of anxiety-like behavior (Figure 6).

Previous studies also demonstrated that alpha-CaMKII (+/-) heterozygous knockout mice displayed aggressive behaviors towards to their cage mates [16,17]. Further investigation demonstrated that these mice displayed an increase in defensive aggression but displayed normal offensive aggression [16], which seems to reflect a decrease in the fear response observed in these mice [16]. Interestingly, we also observed that alpha-CaMKII overexpressing mice exhibit a high frequency of fighting in their home cages, suggesting that both up-regulation and down-regulation of alpha-CaMKII leads to an increase in aggressive behavior. To answer why both groups of mice display similarities of aggressive behavior, we analyzed the aggressive behavior of the alpha-CaMKII overexpressing mice using the resident-intruder test. In contrast to the observations in alpha-CaMKII (+/-) heterozygous knockout mice, alpha-CaMKII overexpressing mice exhibited an increase in offensive aggression but displayed normal defensive aggression. This increase in offensive aggression is thought to reflect the increase in anxiety observed in alpha-CaMKII overexpressing mice (Figure 2). Therefore, the comparison of phenotypes between alpha-CaMKII (+/-) heterozygous knockout and overexpression mice indicates that both an increase and decrease in expression levels of alpha-CaMKII leads to an increase in aggressive behavior, but the aggression state is different between up-regulation and down-regulation of alpha-CaMKII expression levels in the forebrain; up-regulation and down-regulation of alpha-CaMKII levels increases offensive and defensive aggression, respectively (Figure 6). These changes in the aggression state seem to reflect opposite anxiety states between the mice up-regulating and down-regulating the expression levels of alpha-CaMKII.

Previous studies have pointed out that alpha-CaMKII (+/-) heterozygous knockout mice display behavioral abnormalities similar to the symptoms observed in patients with psychiatric diseases such as schizophrenia and bipolar disorders [17]. In this study, we observed that alpha-CaMKII overexpressing mice displayed an increase in anxiety-like behaviors. Comparison between this and previous studies suggest that changes in alpha-CaMKII expression levels strongly affects the emotional state; up-regulation and down-regulation of CaMKII causes a increase or decrease, respectively, in anxiety-like behavior. Therefore, it is possible that the abnormal alteration of alpha-CaMKII expression levels is implicated in psychiatric disorders. It is important to investigate the molecular mechanisms responsible for the regulation of alpha-CaMKII expression in vivo and identify the factors that affect the expression levels of alpha-CaMKII.

## Conclusion

In summary, we generated transgenic mice overexpressing alpha-CaMKII in the forebrain and showed that these transgenic mice displayed an increase in anxiety-like behaviors and offensive aggression; both of which are in contrast to the emotional behavior phenotypes observed in alpha-CaMKII (+/-) heterozygous knockout mice. From these findings, we suggest that the expression levels of alpha-CaMKII positively correlate with the anxiety state and strongly affect aggression state. Therefore, changes in the expression levels of alpha-CaMKII might be associated with psychiatric disorders such as anxiety, depression and bipolar disorders.

## Methods

### Animals

Mice were housed in cages of five or six, maintained on a 12 h light/dark schedule and allowed *ad libitum *access to food and water in their home cages. All experiments were conducted during the light phase of the cycle in an illuminated testing room, according to the *Guide for the Care and Use of Laboratory Animals*, Japan Neuroscience Society, and Tokyo University of Agriculture. Mice were at least 8 weeks of age when tested. All experiments were conducted blind to the treatment condition of the mouse. Animal behavior was recorded using a video camera.

### Generation of transgenic mice

To introduce *Mlu*I restriction sites at the 5' and 3' ends of the cDNA encoding alpha-CaMKII, a plasmid containing wild-type CaMKII (kindly provided by Dr. Alcino J. Silva) was amplified by PCR with the following primers: forward primer, GGGGATATCGCCGCCACCATGGACTACAAGGACGACGATGACAAACTCAAAACGCGTGCTACCATCACCTGCACCCGATTC, and reverse primer, GGGGATATCACGCGTTCAATGCGGCAGGACGGA. The resulting fragment was ligated with a 12.5 kb *Mlu*I fragment containing the alpha-CaMKII promoter, a hybrid intron in the 5' untranslated leader sequence, Kozak consensus sequence, FLAG-tag sequence following an initiation codon and the SV40 polyadenylation signals from pMM-403-CaMKIV [18,19], generating a new construct, pMM-403-alpha-CaMKII. The transgene contained an alpha-CaMKII promoter, a hybrid intron in the 5' untranslated leader sequence, the coding region of alpha-CaMKII and a SV40 polyadenylation [poly (A)] signal. pMM-403-alpha-CaMKII was digested with *Sfi*I and transgenic mice were generated by injecting the purified insert into the pronuclei of C57BL/6N mice (Charles River, Kanagawa, Japan). Genotyping was performed by Southern blot analysis using a probe derived from a 1.1 kb fragment produced by *Eco*RV/*Not*I digestion [SV40 poly (A) probe] from pNN-265-LBDG521R-CREBS133A [18].

### *In situ *hybridization analysis

*In situ *hybridization was preformed as previously described [26,27]. Brains were frozen on dry ice and sectioned on a cryostat at 16 μm. The sections were fixed in 4% paraformaldehyde in phosphate-buffered saline (PBS) and washed in PBS containing 0.1% diethylpyrocarbonate. After two washes in 5 × saline sodium citrate (SSC), the sections were incubated in prehybridization solution [50% formamide, 5 × SSC, 2% DNA-blocking solution (Roche Diagnostics, Mannheim, Germany) and 40 μg/ml salmon testis DNA] for 2 h at 58°C and hybridized in the above prehybridization solution containing digoxigenin (DIG)-labelled anti-sense RNA probes overnight at 58°C. After washes in 2 × SSC and 0.1 × SSC at 65°C, the sections were rinsed in antibody buffer (100 mM Tris pH 7.5, 150 mM NaCl and 0.5% DNA-blocking solution) and incubated in antibody buffer containing a 1:5000 dilution of anti-DIG antibody coupled with alkaline phosphatase (AP) (Roche Diagnostics) for 2 h at room temperature. After two washes in antibody solution and a wash in Tris-buffered saline (TBS) buffer containing 100 mM Tris pH 9.5, 100 mM NaCl and 50 mM MgCl_2_, the sections were stained in TBS buffer containing nitroblue tetrazolium and 5-bromo-4-chloro-3-indoryl phosphate (Roche Diagnostics) overnight at room temperature. The sections were rinsed in water and 95% ethanol and mounted. Control experiments were performed using sense DIG-labeled probes. Sense and anti-sense probes were generated from the 1.1 kb *Eco*RV-*Not*I fragment of pNN-LBDG521R-CREBS133A cloned into pBluescriptII-SK (Stratagene, CA) using an *in vitro *transcription kit (Ambion, TX).

### Quantitative real-time polymerase chain reaction (qRT-PCR)

qRT-PCR was performed in an ABI PRISM 7000 with SYBR green PCR master mix (Applied Biosystems, CA) according to the manufacturer's protocol. Amplification of the single PCR product was confirmed by monitoring the dissociation curve and electrophoresis on 1.2% agarose gels stained with ethidium bromide. The reaction was first incubated at 50°C for 2 min, then at 95°C for 10 min, followed by 40 cycles of 95°C for 15 sec and 60°C for 1 min. All measurements were performed in triplicate. Levels of GAPDH mRNA was used to normalize the relative expression levels of target mRNA. The PCR primers used were as follows (5' to 3'): Tdo2 forward, CTATGAGTGGGTGCCCGTTT; Tdo2 reverse, CCTCCTTTGCTGGCTCTGTT; Dsp forward, GCCGTCAAAATCACCAACC; Dsp reverse, CCATCCAGCACATCCCTCT; Npnt forward, CGCTATGGAGGCAGGATTG; Npnt reverse, TCCGTGTTTGCACTGTGGTT; Pnck forward, ATGGTCTCTGACTTTGGCCTGT; Pnck reverse, TTCCCGTAGGGTTTCTGCTC; Slc39a6 forward, CAGCAAGTGAGAAGAAGGCAGA; Slc39a6 reverse, ACCAAGATGACTCCCAGCAGA; GAPDH forward, ATGGCCTTCCGTGTTCCTAC; GAPDH reverse, GCCTGCTTCACCACCTTCTT.

### Western blot analysis

Samples of the hippocampus and cortex of alpha-CaMKII and WT littermate control mice were isolated, homogenized in SDS buffer (0.1 M dithiothreitol (DTT), 2% SDS and 0.05 M Tris, pH 6.8), heated at 95°C for 10 min and centrifuged at 4°C for 10 min. The supernatants (50 μg of total protein) were analyzed by western blotting. Western blotting, using an anti-alpha-CaMKII antibody (1:2000; Santa Cruz Biotechnology, CA), anti-phospho-alpha-CaMKII T286 antibody (1:2000 Promega, WI), and an anti-alpha-tubulin antibody (1:1000; Santa Cruz Biotechnology) were performed as previously described [28]. Positive antibody binding was visualized using ECL chemiluminescence (GE Healthcare Bio-Sciences Corp., Piscataway, NJ), and membranes were analyzed with the Lumi-imager TM chemiluminescence detection system (Roche Diagnostics, Basel, Switzerland). The amount of protein in lanes was confirmed to be comparable by reprobing with an anti-alpha-tubulin antibody.

### Behavioral procedures

#### Open field test [29]

Mice were placed into the center of a square open field chamber (40 cm long × 40 cm wide × 40 cm high) that was surrounded by white acrylic walls. The total length of the path the mouse traveled (locomotor activity) and the time it spent in a center square (24 cm × 24 cm; % center) were measured over the course of 5 min using an automatic monitoring system (TARGET 2, Neuroscience Inc., Tokyo, Japan).

#### Elevated zero maze test [29]

The zero maze consisted of a circular path (5.5 cm width, inner diameter of 46 cm) that had two open and two closed sections (wall was 15 cm high) and was elevated 50 cm above the floor. Mice were initially placed in the closed section and their behavior was observed for 5 min. The length of time that they spent in the open section (open section time) and the number of times they entered into the open section with two or four paws (number of entries into open section) were measured.

#### Light/dark transition test

The apparatus used for the light/dark transition test consisted of a box (21 × 42 × 25 cm) divided into two sections of equal size by a partition with a door (O'Hara & Co., Ltd., Tokyo, Japan). One box is brightly illuminated by white diodes (390 lux), whereas the other box is dark (2 lux). Mice were placed into the dark side and the door was opened 3 sec after the mouse was detected by the infrared camera. The door was used so that the mice do not enter the light box immediately after release with their motivation to escape from the experimenter, since the latency to enter the light box may serve as an index of anxiety-like behavior. Mice were allowed to move freely between the two boxes for 5 min. The distance traveled in each box, the number of transitions, the time spent in each box and the latency to enter the light box were measured by Image LD4 (O'Hara & Co., Ltd.), modified software based on the public domain NIH Image program (developed at the U.S. National Institutes of Health and available on the Internet at ).

#### Social interaction test [19]

Each male adult subject (alpha-CaMKII overexpressing mouse or WT littermate) was placed in a measuring cage and allowed to stay for 60 min. A male juvenile was introduced into the cage and the amount of time spent in social interaction (e.g. grooming, licking, sniffing, and crawling over or under) with the tested animal was recorded during a 3 min session.

#### Locomotor activity monitoring in the home cage

Daily locomotor activity in the home cage was detected by 124 infrared photocell beams in an adjustable frame placed over the cage for every individual mouse (Biotex, Kyoto, Japan). The animals were maintained on a 12 hr light: 12 hr dark (LD) lighting cycle after habituation for a week before recording.

#### Rotarod test [29]

Mice were placed on a rotating drum (3 cm in diameter; O'Hara & Co., Ltd.). The drum was initially rotated at a speed of 4 rpm after which it was accelerated gradually to 40 rpm over the course of 5 min. The amount of time that the mouse remained on the accelerating rod (running time) was recorded as an indicator of their motor performance, on 3 successive days.

#### Resident-intruder test [29]

To assess offensive aggression, resident mice were individually housed for 4 weeks prior to the introduction of a WT male intruder mouse, which was previously housed in a group of five animals. When we assess offensive aggression, the resident mouse was paired with the same intruder mouse for 3 min. To assess defensive aggression, mice that had previously played the role of the resident animal became the intruder; a different group of WT mice that had been individually housed for 4 weeks, were used as the residents. When we assess defensive aggression, resident mouse was paired with the same intruder mouse for 10 min. The latency to first attack bite (attack latency) and the total number of attacks by the test mice (number of attacks) were measured.

### Statistics

All values are expressed as the mean + SE. Data were analyzed using an analysis of variance (ANOVA) and a single-factor ANOVA and Newman-Keuls *post hoc *comparison were used to analyze differences between groups for the behavioral tests.

## Competing interests

The authors declare that they have no competing interests.

## Authors' contributions

SK is responsible for the hypothesis development and overall design of the research and experiment, and supervised the experimental analyses. SK and SHasegawa co-wrote the manuscript. SHasegawa, TF, TY, KE, MS, TM and RS performed behavioral analyses. TY and RM generated transgenic mice overexpressing alpha-CaMKII. AK constructed a transgene. SHasegawa, TF and KK performed biochemical analyses. SHomma, SM and KY supervised experimental analyses.

## Supplementary Material

Additional file 1 Figure S1. Gene expression analyses in hippocampus of alpha-CaMKII overexpressing mice. (A-E) The mRNA expression levels of nephronectin (A), tryptophan 2,3-dioxygenase (B), solute carrier family 39 (metal ion transporter), member 6 (C), desmoplakin (D) and pregnancy upregulated non-ubiquitously expressed CaM kinase (E) in the hippocampus of alpha-CaMKII overexpressing (n = 5) and WT mice (n = 5). The asterisk indicates statistical significance at P < 0.05. Click here for file
