# Supplementary figures and images for: Transgenic up-regulation of alpha-CaMKII in forebrain leads to increased anxiety-like behaviors and aggression
Source: Mol Brain. 2009 Mar 4;2:6. doi: 10.1186/1756-6606-2-6 (PMC2660323; doi:10.1186/1756-6606-2-6)

**Figure S1**

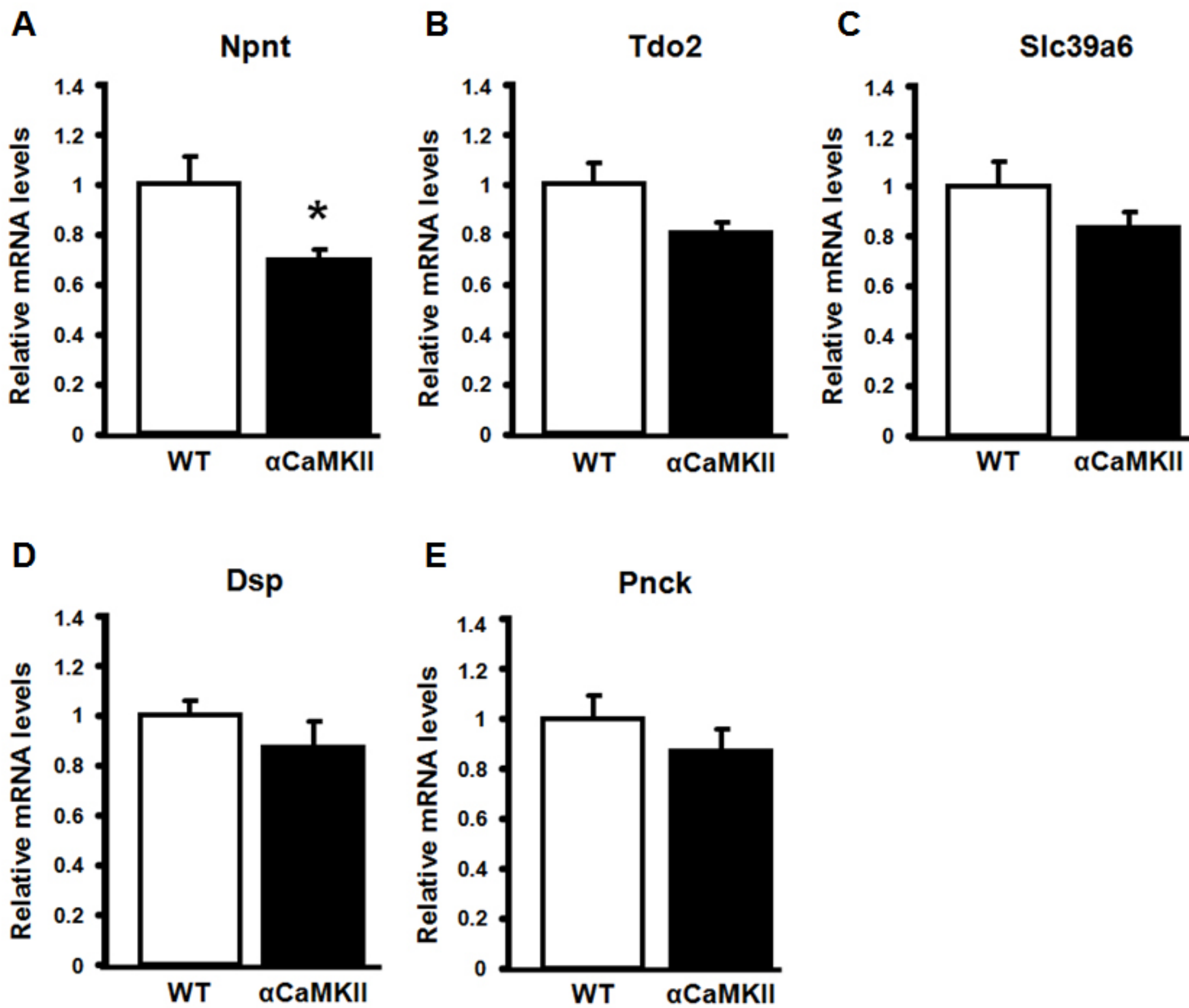

Supplement: Additional file 1 — Figure S1. Gene expression analyses in hippocampus of alpha-CaMKII overexpressing mice. (A-E) The mRNA expression levels of nephronectin (A), tryptophan 2,3-dioxygenase (B), solute carrier family 39 (metal ion transporter), member 6 (C), desmoplakin (D) and pregnancy upregulated non-ubiquitously expressed CaM kinase (E) in the hippocampus of alpha-CaMKII overexpressing (n = 5) and WT mice (n = 5). The asterisk indicates statistical significance at P < 0.05. [file 1756-6606-2-6-S1.pdf]
